# A ferroptosis-related gene signature for overall survival prediction and immune infiltration in lung squamous cell carcinoma

**DOI:** 10.1042/BSR20212835

**Published:** 2022-08-31

**Authors:** Ti-wei Miao, De-qing Yang, Fang-ying Chen, Qi Zhu, Xin Chen

**Affiliations:** 1 Department of Integrated Traditional Chinese and Western Medicine, Zigong First People’s Hospital, Zigong, China; 2 Respiratory Group, Department of Integrated Traditional Chinese and Western Medicine, West China Hospital of Sichuan University, Chengdu, China; 3 Department of Pharmacy, The Second Affiliated Hospital of Kunming Medical University, Kunming, China; 4 Department of Tuberculosis, The Third People’s Hospital of Tibet Autonomous Region, Lhasa, China

**Keywords:** bioinformatics, chemotherapy., ferroptosis-related genes, immunotherapy, lung squamous cell carcinoma, survival

## Abstract

Background: Ferroptosis is associated with cancer initiation and progression. However, the molecular mechanism and prognostic value of ferroptosis-related genes in lung squamous cell carcinoma (LUSC) are poorly understood.

Methods: The mRNA expression profiles, methylation data, and clinical information of patients with LUSC were downloaded from TCGA and GEO database. Ferroptosis-related differentially expressed genes (DEGs) were identified between cancerous and non-cancerous tissues, and their prognostic value was systemically investigated by bioinformatic analyses.

Results: A ferroptosis-related gene signature (*ALOX5, TFRC, PHKG2, FADS2, NOX1*) was constructed using multivariate Cox regression analysis and represented as a risk score. Overall survival (OS) probability was significantly lower in the high-risk group than in the low-risk group (*P*<0.001), and receiver operating characteristic curve showed a good predictive capacity (AUC = 0.739). The risk score was an independent prognostic factor for LUSC. A nomogram was constructed to predict the OS probabilities at 1, 3, and 5 years. High-risk score was associated with increased immune infiltration, lower methylation levels, higher immune checkpoint genes expression levels, and better chemotherapy response. Cell adhesion molecules, focal adhesion, and extracellular matrix receptor interaction were the main pathways in the high-risk group. The signature was validated using the TCGA test cohort, entire TCGA cohort, GSE30219, GSE157010, GSE73403, and GSE4573 datasets. The gene disorders in patients with LUSC were validated using real-time PCR and single-cell RNA sequencing analysis.

Conclusions: A ferroptosis-related gene signature was constructed to predict OS probability in LUSC. This could facilitate novel therapeutic methods and guide individualized therapy.

## Introduction

Lung cancer is a common malignancy and the leading cause of cancer-associated deaths worldwide [1]. Non-small cell lung carcinoma (NSCLC) is the most common type of lung cancer and represents approximately 80–85% of all lung cancers [2,3]. Lung squamous cell carcinoma (LUSC) is a subtype of NSCLC and accounts for 30% of lung cancer diagnoses [4,5]. Various genetic and epigenetic changes have occurred in different subtypes of lung cancer [6]. Moreover, in patients with lung adenocarcinoma (LUAD), targeted therapies (against EGFR, ALK, ROS1, or BRAF) have significantly improved the clinical outcome [7]. However, these effective therapeutic methods may not be suitable for patients with LUSC [6]. Meanwhile, surgical intervention and chemotherapy and/or radiation have been proven to be suitable against a part of LUSC [8]. The 5-year survival rate of lung cancer is approximately 18% [9]. Therefore, considering the limited therapeutic strategies and prognostic models, a systematic study to explore the differentially expressed genes (DEGs) is required to identify a prognostic signature and guide decision-making for LUSC treatment.

Ferroptosis is described as a non-apoptotic regulated cell death, which is an iron-dependent form induced by erastin, characterized by excess reactive oxygen species (ROS) generation and lipid peroxidation [10]. Ferroptosis has been found to occur in lung cancer in recent years [11]. An increased retention of P53 in the nucleus is induced by the cytosolic P53RRA-Ras GTPase-activating protein-binding protein 1 (G3BP1) interaction, which triggers cell cycle arrest, apoptosis, and ferroptosis [12]. Thus, suppressing ferroptosis could inhibit lung cancer cell growth and migration [13]. *SLC7A11*, a ferroptosis-related gene, can repress the progression of NSCLC by ferroptosis-associated pathways [14]. However, whether these ferroptosis-related genes are altered in LUSC and correlated with LUSC patient prognosis remain largely unknown.

In the present study, the mRNA expression profiles, methylation data, and clinical information of patients with LUSC were obtained from The Cancer Genome Atlas (TCGA) and Gene Expression Omnibus (GEO) database. A prognostic signature was then constructed using ferroptosis-related DEGs and validated in multiple test cohorts. The correlations of risk score with immune infiltration, methylation levels, and immunotherapy and chemotherapy response were evaluated using R language. Finally, real-time PCR and single-cell RNA sequencing analysis (scRNA-seq) were performed to validate the expression levels of five genes in LUSC.

## Materials and methods

### Data collection

The mRNA expression profiles of 502 LUSC samples and 49 control samples, DNA methylation data of 370 LUSC samples, and the clinical information of 504 patients with LUSC were obtained from TCGA database (https://portal.gdc.cancer.gov/). GSE30219, GSE157010, GSE73403, and GSE4573 datasets were obtained from the GEO database (https://www.ncbi.nlm.nih.gov/gds/), which included 293, 235, 69, and 130 patients with lung cancer, respectively. The inclusion criteria were patients with (1) LUSC, (2) mRNA expression profiles, and (3) complete follow-up data. Thus, a total of 490 patients from TCGA, 61 patients from GSE30219, 235 patients from GSE157010, 69 patients from GSE73403, and 130 patients from GSE4573 were included in the present study. The patients from TCGA were randomly divided into training and test cohorts in a 7:3 ratio using ‘glmnet packages’ in R language (version 4.0.2) [15,16]. Thus, 346 and 144 patients were allocated to the TCGA training and test cohorts, respectively. GSE30219, GSE157010, GSE73403, and GSE4573 datasets were considered as the external validation cohort. The baseline characteristics of patients with LUSC are shown in Table 1. The raw data and phenotype data of the GSE111907 dataset were downloaded from the GEO database, which is a scRNA-seq dataset and include 185 samples. The inclusion criteria were (1) LUSC and (2) cancer cell or immune cell. Thus, 12 cancer cell samples and 12 immune cell samples were included in the current study. Sixty ferroptosis-related genes were retrieved from previous literature [17–20].

**Table 1:** Clinical features of patients with LUSC from TCGA and GEO databases

Clinical features	TCGA training cohort (346)	TCGA test cohort (144)	GSE30219 (61)	GSE157010 (235)	GSE73403 (69)	GSE4573 (130)
**Age (years)**						
≥65	226 (65.32%)	94 (65.28%)	27 (44.26%)	156 (66.38%)	25 (36.23%)	78 (60.00%)
<65	119 (34.39%)	50 (34.72%)	34 (55.74%)	79 (33.62%)	44 (63.77%)	52 (40.00%)
Unknown	1 (0.29%)	0	0	0	0	0
**Gender**						
Men	256 (73.99%)	106 (73.61%)	56 (91.80%)	153 (65.11%)	65 (94.20%)	82 (63.08%)
Women	90 (26.01%)	38 (26.39%)	5 (8.205)	82 (34.89%)	4 (5.80%)	48 (36.92%)
Unknown	0	0	0	0	0	0
**T classification**						
T1-T2	276 (79.77%)	121 (84.03%)	55 (90.16%)	202 (85.96%)	46 (66.67%)	109 (83.85%)
T3-T4	70 (20.23%)	23 (15.97%)	6 (9.84%)	31 (13.19%)	23 (33.33&)	21 (16.15%)
Unknown	0	0	0	2 (0.85%)	0	0
**N classification**						
N0	211 (60.98%)	101 (70.14%)	52 (85.25%)	/	35 (50.72%)	83 (63.85%)
N1-N3	132 (38.15%)	40 (27.78%)	9 (14.75%)	/	34 (49.28%)	47 (36.15%)
Unknown	3 (0.87%)	3 (2.08%)	0	/	0	0
**M classification**						
M0	286 (82.66%)	116 (80.56%)	61 (100.00%)	/	69 (100.00%)	129 (99.23%)
M1	6 (1.73%)	1 (0.69%)	0	/	0	0
Unknown	54 (15.61%)	27 (18.75%)	0	/	0	1 (0.77%)
**UICC stage**						
Stage I-II	273 (78.90%)	123 (85.42%)	57 (93.44%)	/	38 (55.07%)	107 (82.31%)
Stage III-IV	70 (20.23%)	20 (13.89%)	4 (6.56%)	/	31 (44.93%)	23 (17.69%)
Unknown	3 (0.87%)	1 (0.69%)	0	/	0	0

### Identification of ferroptosis-related DEGs

The gene expression matrix of LUSC was obtained using Strawberry Perl (5.32.0.1-64bit), and the expression matrix of ferroptosis-related genes was extracted using R language. The ferroptosis-related DEGs were identified using ‘limma packages’ in R language based on |log2 fold change (FC)| > 0 and adjusted *P* value < 0.05. Volcano plots were constructed using ‘ggplot2 packages’ in R language.

### Identification of prognostic genes and construction of a prognostic signature

Prognostic ferroptosis-related genes were identified using univariate Cox regression analysis with *P*<0.05. The prognostic ferroptosis-related DEGs were screened using ‘venn diagram package’ in R language. A prognostic signature was constructed using multivariate Cox regression analysis according to a linear combination of levels of gene expression multiplied by a regression coefficient (β). The risk score was calculated according to the formula: Risk score = levels of gene 1 relative expression × β1 gene 1 + levels of gene 2 relative expression × β2 gene 2 + … + levels of gene *n* relative expression × β*n* gene *n*. The patients were divided into low-risk and high-risk groups according to the median risk score. Kaplan–Meier survival curve was plotted between the high-risk and low-risk groups and compared using the log-rank test. The predictive value of the signature was assessed using receiver operating characteristic curve (ROC) analysis. Principal component analysis (PCA) and t-distributed stochastic neighbor embedding (t-SNE) analysis were performed to explore the distribution position of the two groups. Univariate and multivariate Cox regression analyses were used to screen independent prognostic factors for LUSC.

### Nomogram and calibration plots of the nomogram

A nomogram could better predict the disease prognosis due to its multidimensional parameters [21,22]. A nomogram was constructed using independent prognostic factors (age, UICC stage, and risk score) to predict the OS probability at 1-, 3-, and 5-years using the ‘rms package’ in R language. Calibration plots of the nomogram were applied to check the conformity of the nomogram-predicted and actual OS probabilities.

### Gene set enrichment analysis

Gene set enrichment analysis is a computational method that determines whether a priori defined set of genes shows statistically significant and concordant differences between two biological states [23]. The ‘c2.cp.kegg.v7.5.1.symbols.gmt’ was downloaded from the GSEA database (http://www.gsea-msigdb.org/gsea/index.jsp). The Kyoto Encyclopedia of Genes and Genomes (KEGG) pathway analyses between the high and low-risk groups were performed using ‘limma’, ‘org.Hs.eg.db’, ‘clusterProfiler’, and ‘enrichplot’ packages in R language.

### Analysis of DNA methylation levels of five genes

DNA methylation matrix of LUSC was obtained using Strawberry Perl, and DNA methylation data of five genes (arachidonate 5-lipoxygenase [*ALOX5*], transferrin receptor [*TFRC*], phosphorylase kinase catalytic subunit gamma 2 [*PHKG2*], fatty acid desaturase 2 [*FADS2*], and NADPH oxidase 1 [*NOX1*]) in LUSC were extracted using R language and compared between the high- and low-risk groups using Mann–Whitney test.

### Analysis of immune infiltration and immunotherapy

The mRNA expression matrix of LUSC was converted to the tumor micro-environment score matrix using R language. The tumor micro-environment score including stromal score, immune score, and estimate score were compared between the high and low-risk groups using R language. The correlations of risk score and five genes with different immune cells were determined using R language. Spearman’s rank correlation test was used to analyze the correlations, and the significance level was set at *P*<0.05. Moreover, levels of immune checkpoints genes (*PD1, PDL1, CTLA4*) relative expression were compared between the two risk groups using GraphPad Prism (version 7.00).

### Analysis of sensitivity of chemotherapy drugs

Six chemotherapy drugs including bexarotene [24,25], dasatinib [26,27], embelin [28], midostaurin [29], pazopanib [30–32], and pyrimethamine [33,34] were screened from previous literature, which have been shown to have effects on lung cancer. The half inhibitory concentration (IC50) of these chemotherapy drugs was compared between both risk groups using ‘pRRophetic packages’ in R language. Wilcoxon signed-rank test was used to compare the differences between the two risk groups. *P*<0.05 was considered statistically significant.

### Real-time PCR validation

Ten paired lung tissue samples were obtained from patients with LUSC who underwent lobectomy in West China Hospital of Sichuan University. Histologically normal tissues were considered as controls. Total RNA was extracted using the E.Z.N.A. HP Total RNA Kit (OMEGA, U.S.A.), according to the manufacturer’s instructions. Complementary DNA (cDNA) was synthesized using PrimeScript TM RT reagent Kit (Takara, Japan) following the manufacturer’s instructions. Real-time PCR was performed using Iq TM SYBR Green Supermix (BIO-RAD, U.S.A.) according to the manufacturer’s protocol. Relative expression levels of five genes were normalized by the *β-actin* *C*t value (endogenous reference), applying a 2^−ΔΔ*C*t^ *C*t relative quantification method. The real-time PCR primers were as follow:

*ALOX5*-forward: 5′-CAAAATCTGGGTGCGTTCCA-3′

*ALOX5*-reverse: 5′-AGCAGCTTGAAAATGGGGTG-3′

*TFRC*-forward: 5′-GGAGTGCTGGAGACTTTGGA-3′

*TFRC*-reverse: 5′-TATACAACAGTGGGCTGGCA-3′

*PHKG2*-forward: 5′-AGCTTCGAGAGTTGTGTGGG-3′

*PHKG2*-reverse: 5′-TAACATCAGGATCTGCCGCC-3

*NOX1*-forward: 5′-GGGGTCAAACAGAGGAGAGC-3′

*NOX1*-reverse: 5′-CTTCTGCTGGGAGCGGTAAA-3′

*FADS2*-forward: 5′-GCCACTTAAAGGGTGCCTCT-3′

*FADS2*-reverse: 5′-TGCTGGTGATTGTAGGGCAG-3′

*β-actin*-forward: 5′-CCACGAAACTACCTTCAACTCC-3′.

*Β-actin* -reverse: 5′-GTGATCTCCTTCTGCATCCTGT-3′.

### Single-cell RNA sequencing analysis

The relative expression levels of five genes (*ALOX5, TFRC, PHKG2, FADS2, NOX1*) were compared between the cancer cells and immune cells.

### Statistical analysis

Statistical analysis was performed using GraphPad Prism or R language. Shapiro–Wilk test was applied to test the data distribution type. Levels of relative gene expression were expressed as median (interquartile range). Mann–Whitney test was used in comparing the differences between two groups. *P*<0.05 was considered statistically significant difference.

## Results

### Identification of ferroptosis-related DEGs

**Figure 1:**
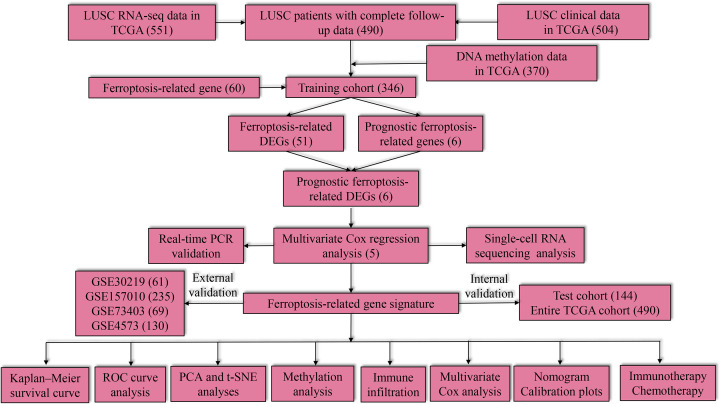
Study flow chart and main findings Numbers within parenthesis indicate the size of the sample obtained.

**Figure 2:**
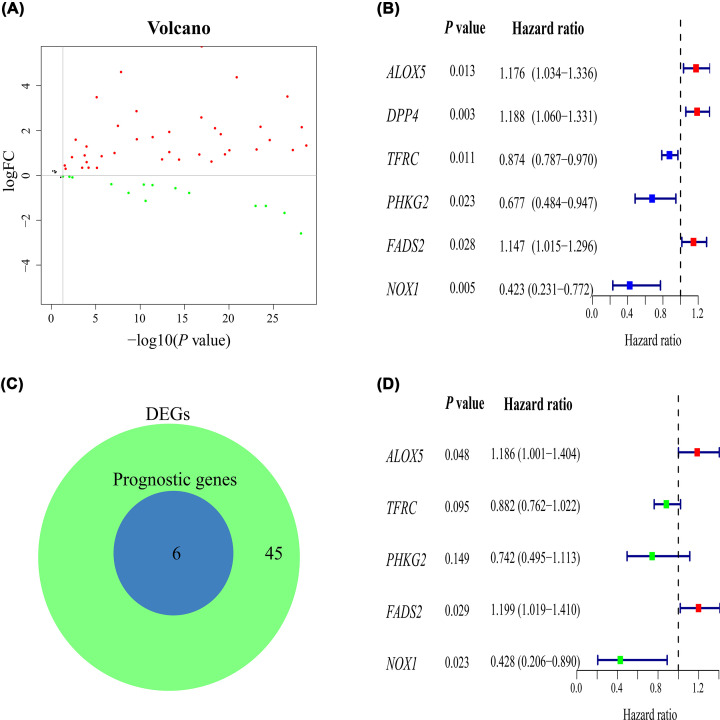
Construction of the prognostic signature (**A**) Volcano plots of ferroptosis-related genes in patients with LUSC versus healthy control. (**B**) Univariate Cox regression analysis. (**C**) The prognostic ferroptosis-related DEGs. (**D**) Multivariate Cox regression analysis. DEG, differentially expressed gene; FC, fold change.

The study flowchart is shown in Figure 1. A total of 51 ferroptosis-related DEGs (38 up-regulated and 13 down-regulated genes) were identified when LUSC was compared with the control (Figure 2A).

### Identification of prognostic ferroptosis-related DEGs

In univariate Cox regression analysis, a total of six ferroptosis-related genes were associated with survival (Figure 2B). There were six prognostic ferroptosis-related DEGs between ferroptosis-related DEGs and prognostic ferroptosis-related genes (Figure 2C).

### Construction of the prognostic signature

**Figure 3:**
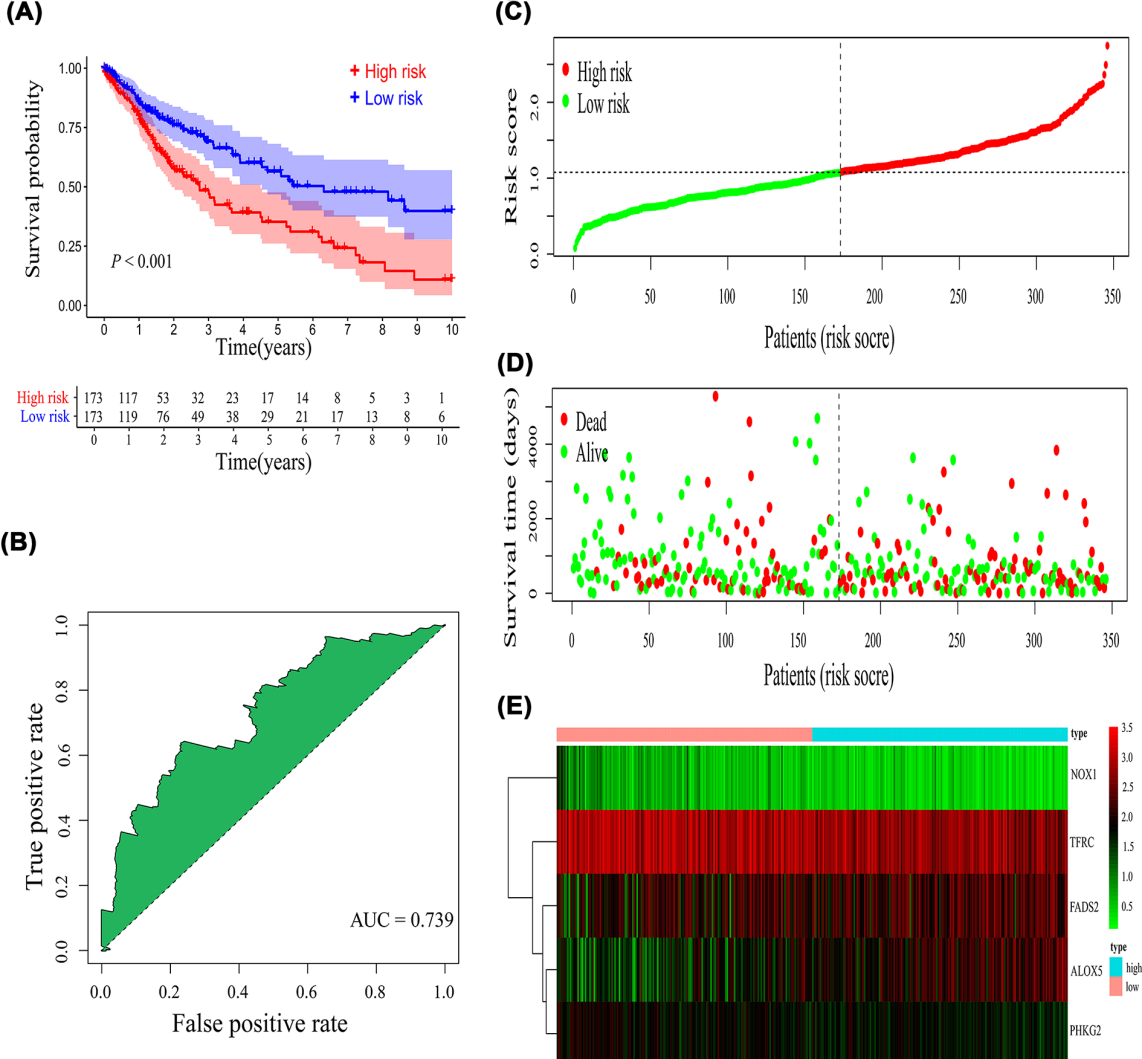
Evaluation of the prognostic signature (**A**) Kaplan–Meier survival curve, (**B**) receiver-operating characteristic curve, (**C**) risk score distribution, (**D**) survival status, and (**E**) heatmap of five gene expression profiles.

**Figure 4:**
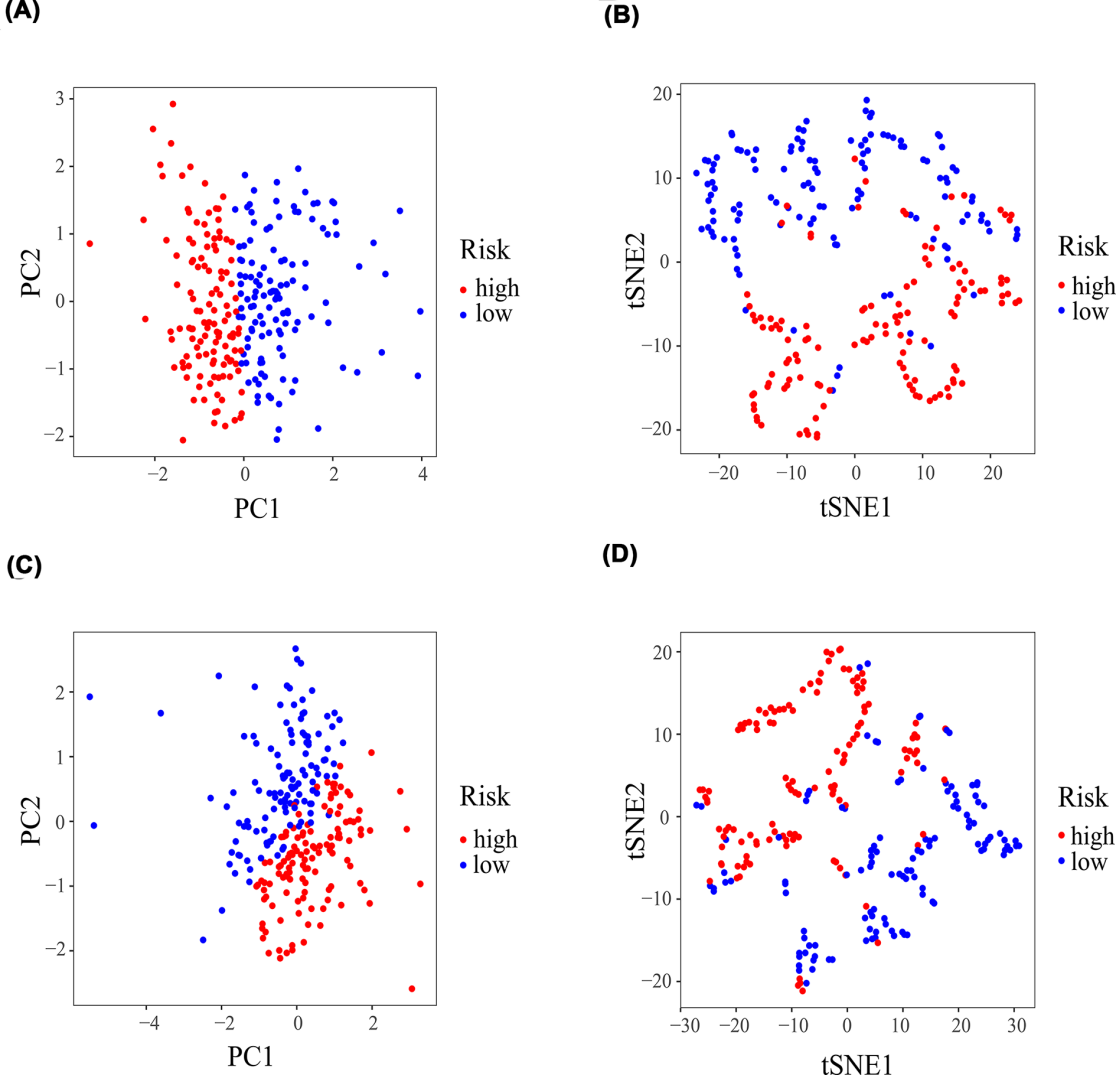
PCA and t-SNE analysis PCA and t-SNE analysis of the prognostic signature in the TCGA (**A,B**) training cohorts and (**C,D**) the test cohorts. PCA, principal component analysis; t-SNE, t-distributed stochastic neighbor embedding.

Multivariate Cox regression analysis was applied to construct the prognostic signature. Subsequently, five genes (*ALOX5, TFRC, PHKG2, FADS2, NOX1*) were screened using R language (Figure 2D), and the risk score formula was established: Risk score = (0.170 × expression of *ALOX5*) + ([−0.125] × expression of *TFRC*) + ([−0.298] × expression of *PHKG2*) + (0.181 × expression of *FADS2*) + ([−0.848] × expression of *NOX1*). The patients with LUSC were stratified into high- or low-risk groups according to the median risk score (Figure 3C). Kaplan–Meier survival curve demonstrated that the high-risk group had a lower OS probability than the low-risk group (*P*<0.001; Figure 3A), and the area under the ROC curve (AUC) value was 0.739 (Figure 3B). Patients in the high-risk group had a higher mortality (Figure 3D). Relative expression levels of the five genes between the two risk groups are shown in a heatmap (Figure 3E). PCA and t-SNE analysis indicated that the patients in the high- or low-risk groups were distributed in different positions (Figure 4A,B).

### Validation of the prognostic signature

**Figure 5:**
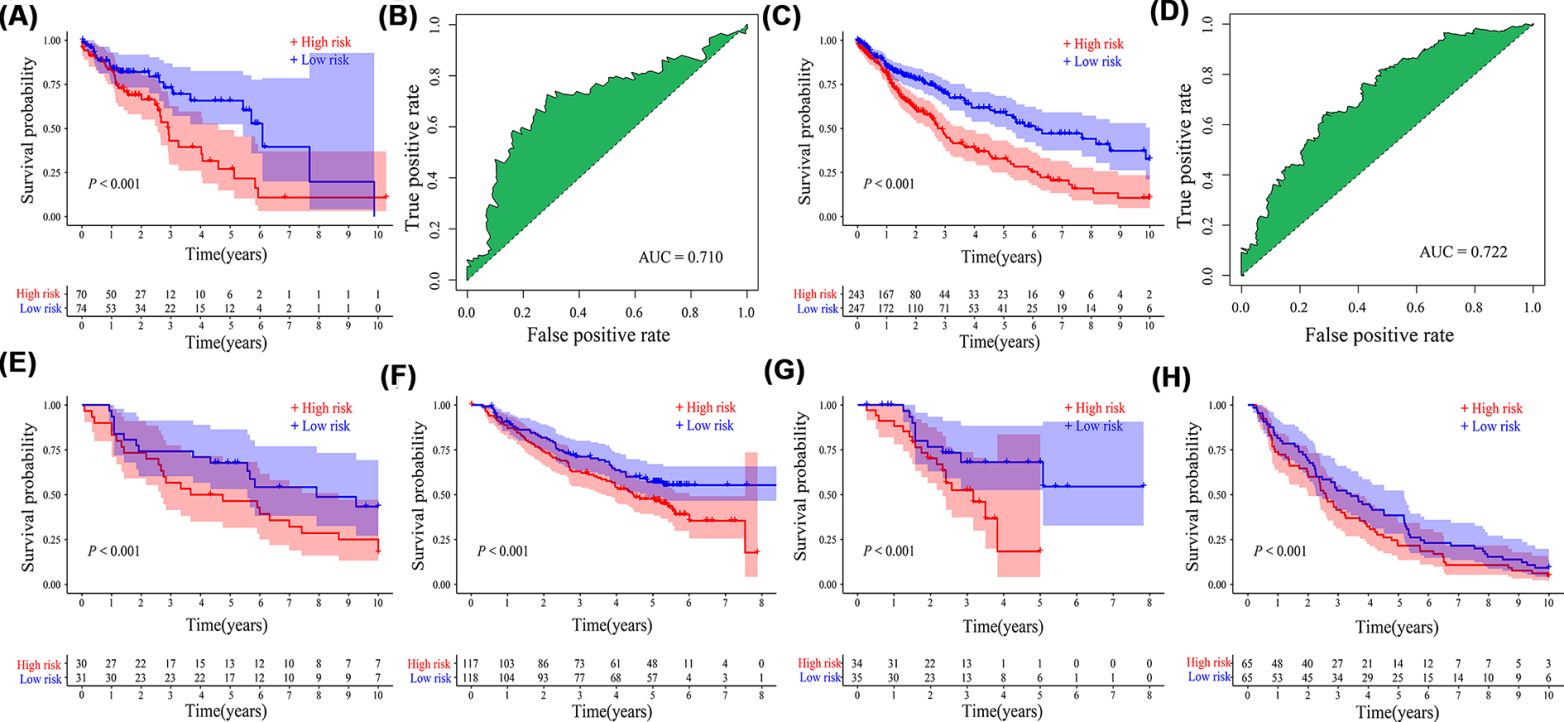
Validation of the prognostic signature Kaplan–Meier survival curve and receiver operating characteristic curve in (**A,B**) the TCGA test cohorts and (**C,D**) the entire TCGA cohorts. Kaplan–Meier survival curve in (**E**) GSE30219, (**F**) GSE157010, (**G**) GSE73403, and (**H**) GSE4573 datasets; AUC, area under the ROC curve.

Kaplan–Meier survival curve showed that OS probability in the high-risk group was significantly lower than that in the low-risk group (*P*<0.001, Figure 5A), and AUC was 0.710 (Figure 5B) in the TCGA test cohorts. In addition, PCA and t-SNE analysis showed that patients in the two risk groups were distributed in different positions (Figure 4C,D). The high-risk group had a lower OS probability compared with the low-risk group (*P*<0.001, Figure 5C), and AUC was 0.722 (Figure 5D) in the entire TCGA cohorts. In addition, the high-risk group also had a lower OS probability than the low-risk group in GSE30219 (*P*<0.001, Figure 5E), GSE157010 (*P*<0.001, Figure 5F), GSE73403 (*P*<0.001, Figure 5G), and GSE4573 (*P*<0.001, Figure 5H).

### Risk score as an independent prognostic factor for LUSC

**Figure 6:**
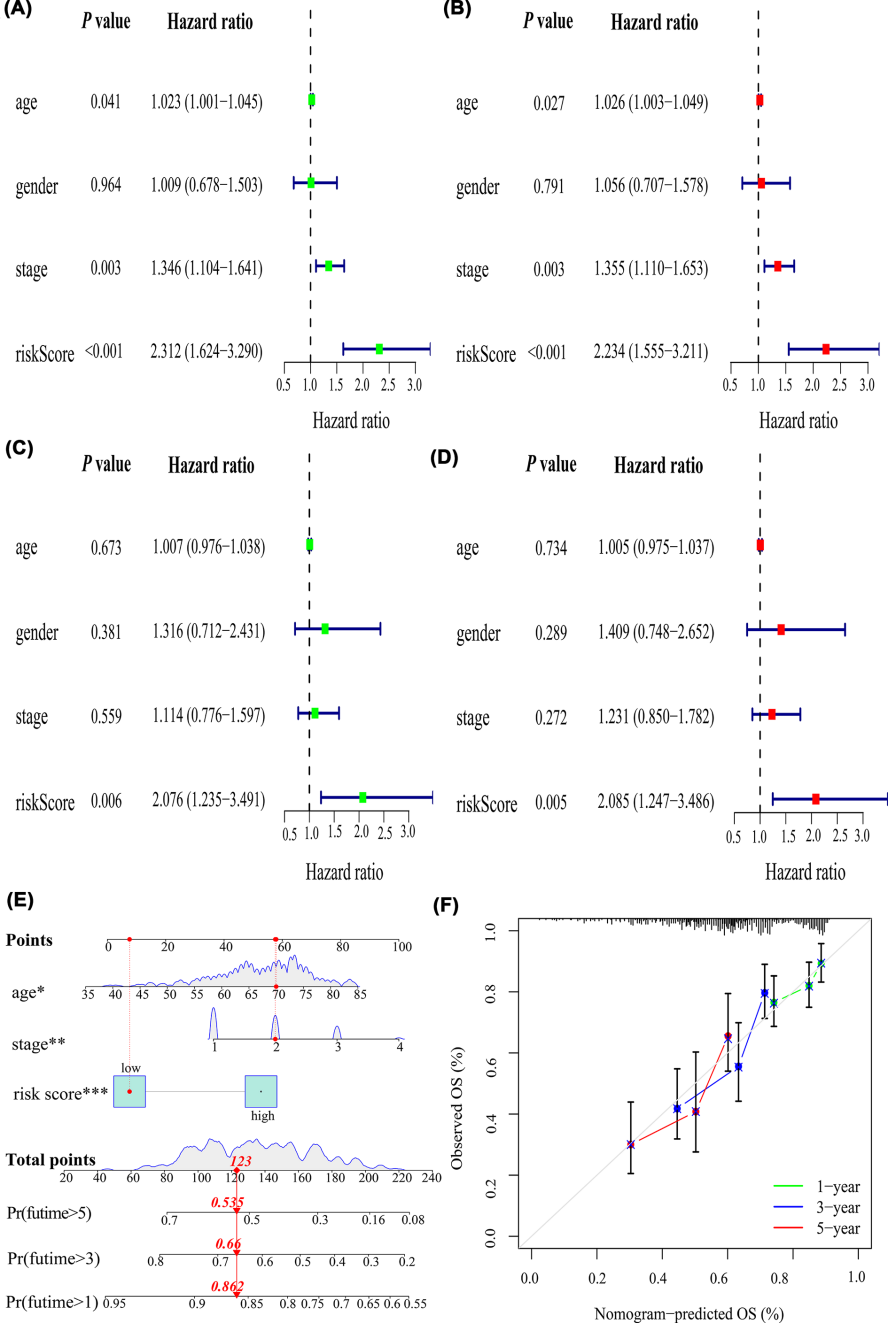
Correlations of the risk score with clinicopathological characteristics and construction of a nomogram and calibration plots Univariable and multivariate Cox regression analysis in the (**A,B**) training cohorts and (**C,D**) test cohorts. (**E**) Nomogram to predict OS at 1, 3, and 5 years. (**F**) Calibration plots of the nomogram. OS: overall survival; *P<0.05, **P<0.01, ***P<0.001.

Age, UICC stage, and risk score were associated with prognosis using univariate Cox regression analysis (hazard ratio [HR]: 1.023, *P*=0.041; HR: 1.346, *P*=0.003; HR: 2.312, *P*<0.001, Figure 6A), which was confirmed using multivariate Cox regression analysis (HR: 1.026, *P*=0.027; HR: 1.355, *P*=0.003; HR: 2.234, *P*<0.001) in the training cohorts (Figure 6B). In contrast, only risk score was associated with prognosis in univariate Cox regression analysis (HR: 2.076, *P*=0.006, Figure 6C) and multivariate Cox regression analysis (HR: 2.085, *P*=0.005, Figure 6D) in the test cohorts. Thus, the risk score was an independent prognostic factor for LUSC.

### Nomogram and calibration plots of the nomogram

A nomogram was successfully constructed to predict the OS probabilities at 1, 3, and 5 years in patients with LUSC, which was calculated by plotting a vertical line between the total point axis and each prognostic axis (Figure 6E). In addition, calibration plots of the nomogram demonstrated high conformity of the nomogram-predicted and actual OS probabilities at 1, 3, and 5 years in patients with LUSC (Figure 6F).

### Gene set enrichment analysis

**Figure 7:**
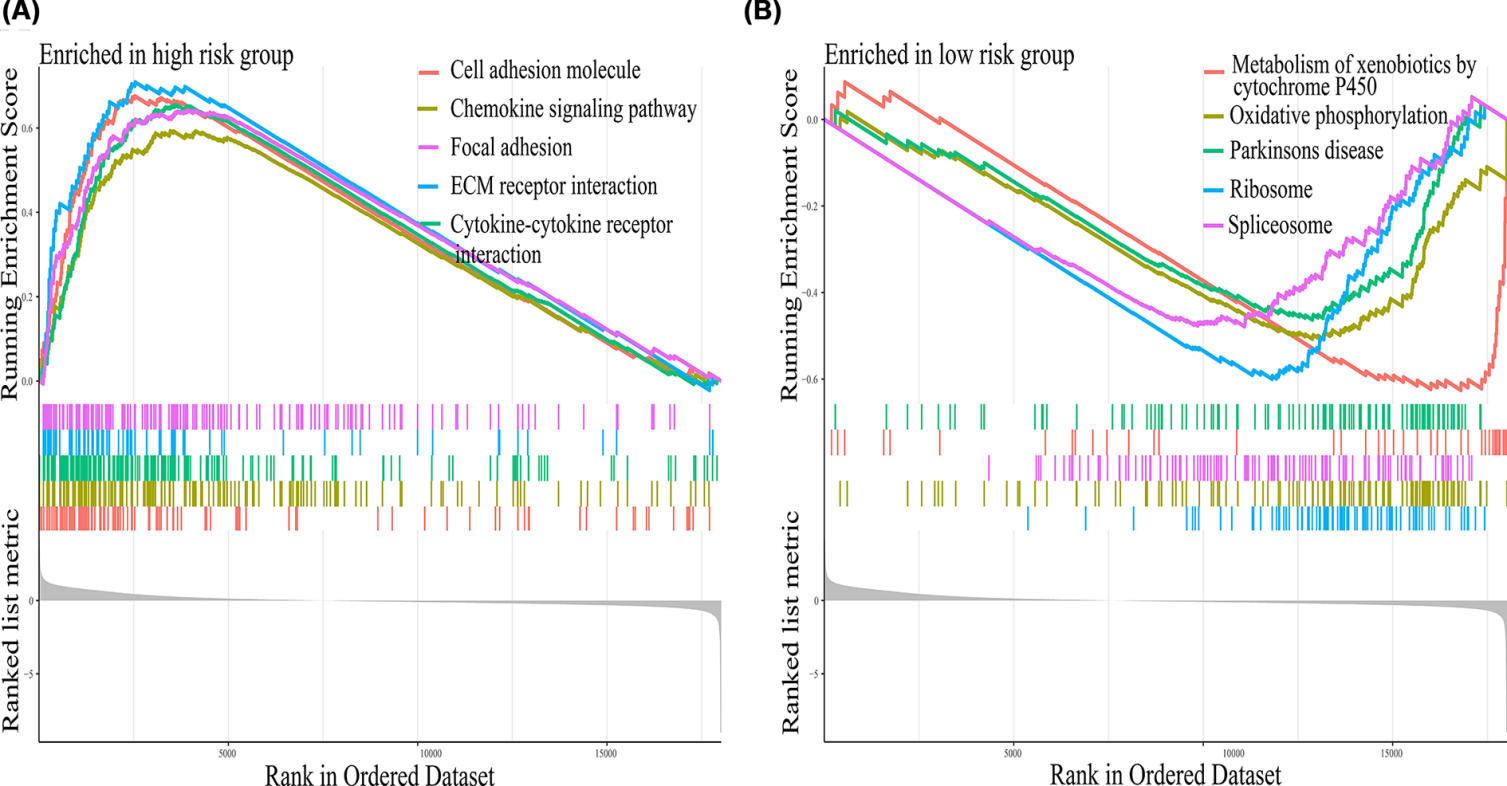
Gene set enrichment analysis The primary pathways enriched in the (**A**) high-risk group and (**B**) low-risk group in LUSC using gene set enrichment analysis.

The results showed that the high-risk group was mainly enriched in cell adhesion molecules, focal adhesion, and extracellular matrix (ECM) receptor interaction (Figure 7A), whereas metabolism of xenobiotics by cytochrome P450, and oxidative phosphorylation were the main pathways in the low-risk group (Figure 7B).

### DNA methylation levels of five genes

**Figure 8:**
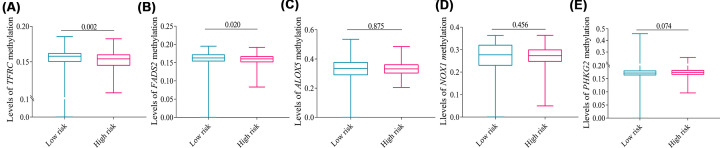
Levels of five genes methylation expression Levels of (**A**) *TFRC*, (**B**) *FADS2*, (**C**) *ALOX5*, (**D**) *NOX1*, and (**E**) *PHKG2* methylation between the two risk groups. Data were expressed as median (interquartile range).

DNA methylation levels of *TFRC* (*P*=0.002) and *FADS2* (*P*=0.020) in the high-risk group were significantly lower than that in the low-risk group (Figure 8A,B), whereas DNA methylation levels of *ALOX5, PHKG2*, and *NOX1* were slightly reduced in the high-risk group compared with the low-risk group without a statistical difference (Figure 8C–E).

### Immune infiltration and immunotherapy

**Figure 9:**
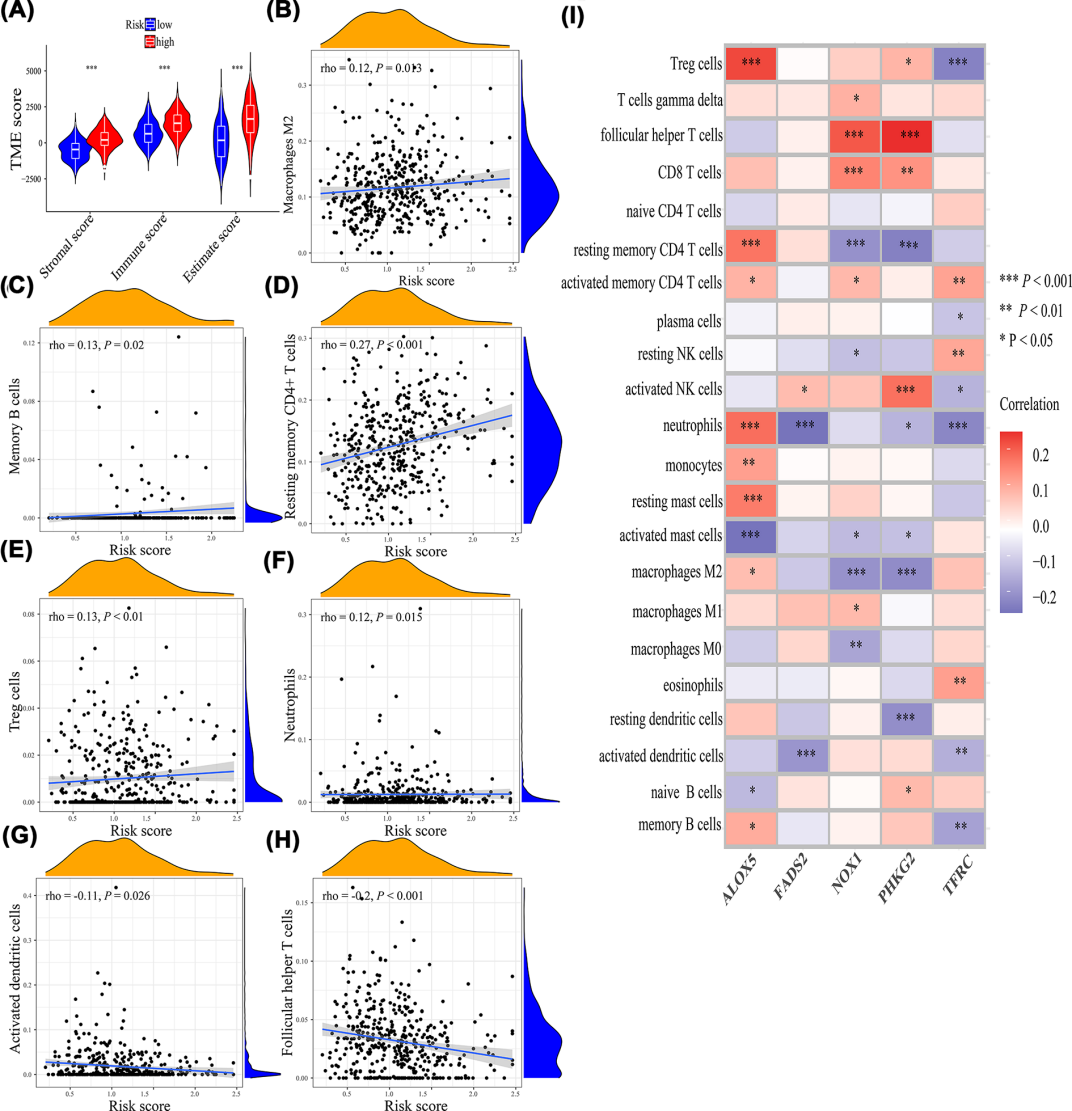
The correlations of risk score with immune infiltration (**A**) Tumor microenvironment score. (**B**) Macrophages M2. (**C**) Memory B cells. (**D**) Resting memory CD4^+^ T cells. (**E**) Treg cells. (**F**) Neutrophils. (**G**) Activated dendritic cells. (**H**) Follicular helper T cells. (**I**) The correlations of five genes with immune infiltration; *P<0.05, **P<0.01, ***P<0.001.

**Figure 10:**
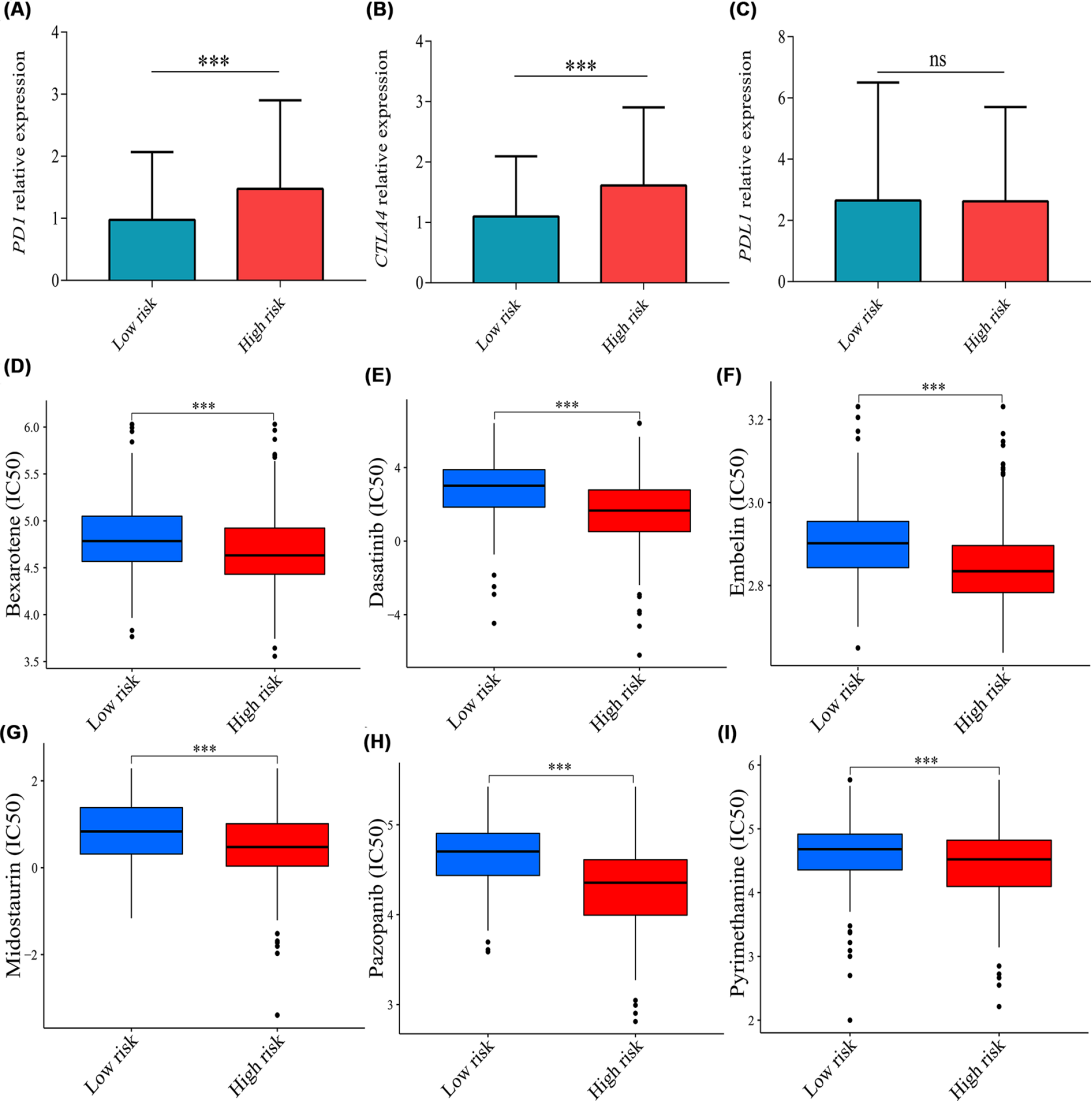
The correlations of risk score with immunotherapy and chemotherapy Levels of (**A**) *PD1*, (**B**) *CTLA4*, and (**C**) *PDL1* relative expression between the high- and low-risk groups. IC50 of (**D**) bexarotene, (**E**) dasatinib, (**F**) embelin, (**G**) midostaurin, (**H**) pazopanib, and (**I**) pyrimethamine between the two risk groups. Data were expressed as median (interquartile range). IC50: half inhibitory concentration; ns, no significance; ***P<0.001.

The tumor microenvironment score including stromal score (*P*<0.001), immune score (*P*<0.001), and estimate score (*P*<0.001) were all higher in the high-risk group compared with the low-risk group (Figure 9A). Thus, the tumor micro-environment was significantly different between the two risk groups. Moreover, the risk score was positively associated with macrophages M_2_ (*P*=0.013, rho = 0.12), memory B cells (*P*=0.02, rho = 0.13), resting memory CD4^+^ T cells (*P*<0.001, rho = 0.27), Treg cells (*P*<0.01, rho = 0.13), and neutrophils (*P*=0.015, rho = 0.12) (Figure 9B–F) and was negatively associated with activated dendritic cells (*P*=0.026, rho = −0.11) and follicular helper T cells (*P*<0.01, rho = −0.2) (Figure 9G,H). The correlations of five genes with immune cells were illustrated in Figure 9I. In addition, to explore the immunotherapy response on different risk groups, relative expression levels of immune checkpoint genes (*PD1, PDL1, CTLA4*) were compared, and the results showed that the relative expression levels of *PD1* (*P*<0.001) and *CTLA4* (*P*<0.001) were higher in the high-risk group (Figure 10A–C), which showed that the high-risk group may have a better immunotherapy response.

### Sensitivity of chemotherapy drugs

The results indicated that the high-risk group exhibited a lower IC50 for bexarotene, dasatinib, embelin, midostaurin, pazopanib, and pyrimethamine (*P*<0.001, Figure 10D–I), suggesting that the prognostic signature may be a reference option for chemotherapy drugs.

### Real-time PCR validation

**Figure 11:**
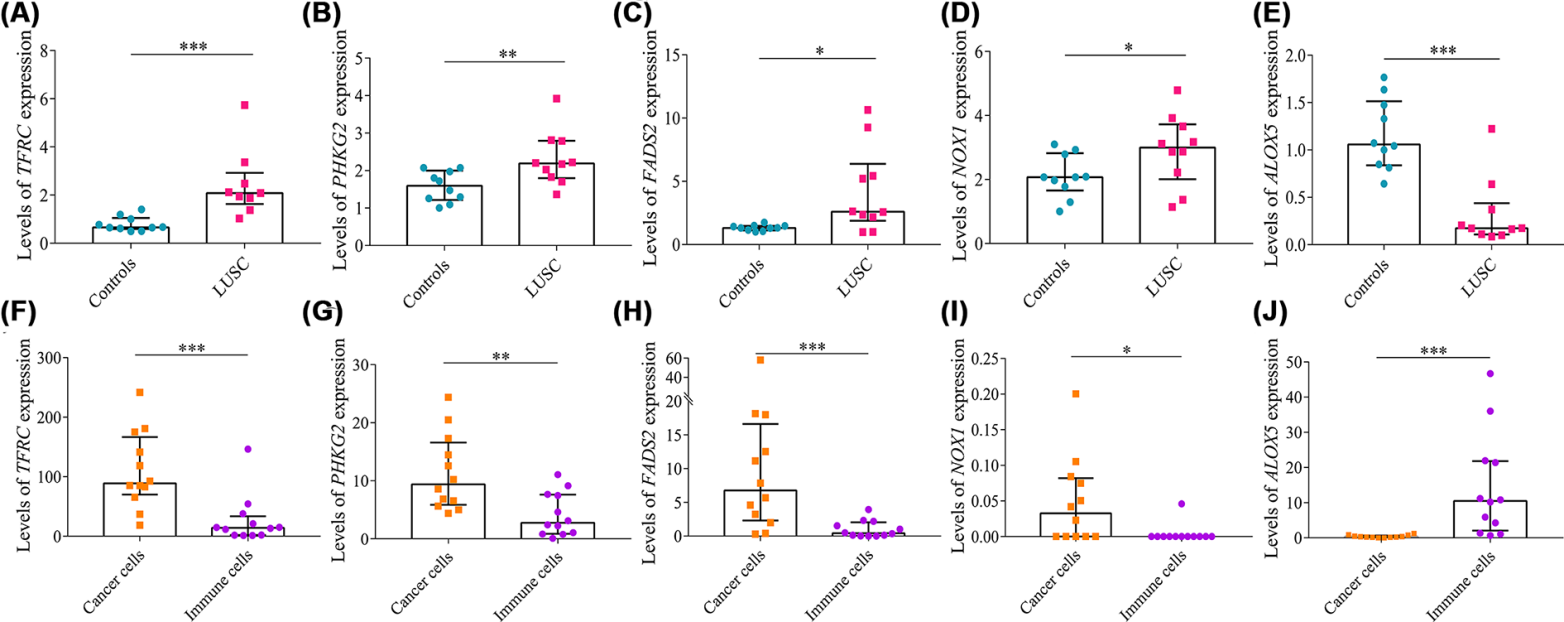
Real-time PCR and single cell RNA sequencing analysis Levels of (**A**) *TFRC*, (**B**) *PHKG2*, (**C**) *FADS2*, (**D**) *NOX1*, and (**E**) *ALOX5* relative expression in lung tissues using real-time PCR. Relative expression levels of (**F**) *TFRC*, (**G**) *PHKG2*, (**H**) *FADS2*, (**I**) *NOX1*, and (**J**) *ALOX5* between cancer cells and immune cells using single cell RNA sequencing analysis. Data were expressed as median (interquartile range); *P<0.05, **P<0.01, ***P<0.001.

Levels of *TFRC* (*P*<0.001), *PHKG2* (*P*=0.008), *FADS2* (*P*=0.023), and *NOX1* (*P*=0.049) mRNA relative expression were higher in tumor tissues than in control tissues (Figure 11A–D); however, levels of *ALOX5* (*P*<0.001) mRNA relative expression were reduced in cancerous tissues compared with control tissues (Figure 11E), which were consistent with the bioinformatic results.

### Single-cell RNA sequencing analysis

Many types of immune cells infiltrate tumors, thus clarifying that the resource of five genes is of great importance. Levels of *TFRC* (*P*<0.001), *PHKG2* (*P*=0.005), *FADS2* (*P*<0.001), and *NOX1* (*P*=0.011) mRNA relative expression were higher in cancer cells than in immune cells (Figure 11F–I), whereas levels of *ALOX5* (*P*<0.001) mRNA relative expression were lower in cancer cells than in immune cells (Figure 11J).

## Discussion

In the present study, a ferroptosis-related gene signature was constructed to predict prognosis in patients with LUSC using multivariate Cox regression analysis, and the risk score was determined to be an independent prognostic factor for LUSC. A nomogram was successfully constructed to predict the OS probabilities at 1, 3, and 5 years in patients with LUSC. The high-risk score was associated with increased immune infiltration, lower methylation levels, higher levels of immune checkpoint genes, and better chemotherapy drugs sensitivity. Finally, the prognostic signature was validated using the TCGA test cohorts, entire TCGA cohorts, and multiple GEO datasets.

*TFRC*, also known as CD71, is an essential membrane protein-regulating intracellular iron transporter [35,36]. Activating *TFRC* increases the iron content, mediates the release of ROS, and induces lipid peroxidation, which further promotes the ferroptosis of cells [37]; however, knocking down *TFRC* significantly inhibits cancer cell proliferation and metastasis via up-regulation of AXIN2 expression or sponge of microRNA-107 [38,39]. Levels of *TFRC* mRNA expression were significantly increased in LUSC samples [40]. PhK is a heterotetramer composed of four copies each of α, β, γ, and ∆ subunits [41]. Subunit γ is encoded by *PHKG2* [42]. Silencing *PHKG2* prevents accumulation of lipid peroxides and decreases cellular iron level [43]. *PHKG2* is a useful diagnostic biomarker for multiple cancers, including breast cancer [44] and endometrial cancer [45]. *NOX1* plays an important role in ROS generation and lung cancer [46,47]. *NOX1*-dependent ROS generation for toll-like receptor 4 (TLR4) signaling is found to enhance the metastasis of NSCLC [48]. In addition, *NOX1* up-regulation is shown to activate sirtuin 1 (SIRT1) and inhibit P53 [49]. *FADS2* is overexpressed in cancer and functions as a potential oncogene that facilitates cancer cell proliferation [50]. Inhibiting *FADS2* could reduce ferroptosis by increasing levels of Fe and lipid ROS in lung cancer cells [51]. *ALOX5* gene encodes lipoxygenase, which could catalyze the conversion of arachidonic acid to leukotriene [52]. Knockdown of *ALXO5* mitigates lipid peroxidation, mitochondrial damage, DNA impairment, and cell death in ARPE-19 cells [53]. Genetic variations in the promoter region of *ALOX5* may induce a reduced drug response to montelukast sodium in patients with asthma, leading to pharmacogene [54]. *ALOX5* polymorphisms in non-smokers may increase risk of lung cancer [55]. The increased *TFRC, PHKG2, FADS2, NOX1,* and reduced *ALOX5* levels in LUSC tissues were reported in the present study, and scRNA-seq analysis showed that the levels of the five dysregulated genes were mainly influenced by cancer cells rather than immune cells.

The ferroptosis-related gene signature has been successfully constructed to predict the prognosis in patients with hepatocellular carcinoma [56], breast cancer [57], pancreatic adenocarcinoma [58], and cholangiocarcinoma [59]. Additionally, in LUAD, a ferroptosis-related gene signature including five genes was constructed to predict prognosis [60]. However, the ferroptosis-related gene signature for LUAD was not suitable for LUSC due to disease heterogenicity [6], different responses to clinical treatment [61,62], different prognoses [63], and lack of an experimental validation. Therefore, in the present study, a ferroptosis-related gene signature was successfully constructed using five genes validated by real-time PCR to predict the prognosis in patients with LUSC. Kaplan–Meier survival curve showed that patients with LUSC in the high-risk group were associated with a lower OS probability with moderate sensitivity and specificity than patients in the low-risk group. Moreover, the risk score was an independent prognostic factor for LUSC through multivariate Cox regression analysis. The signature was successfully validated using the TCGA test cohort, entire TCGA cohort, GSE30219, GSE157010, GSE73403, and GSE4573. Thus, the prognostic signature could be used to discriminate different risk groups and may contribute to guide therapy.

GSEA was performed to explore the underlying molecular mechanism between the two risk groups. The abnormal expression of cell adhesion molecules resulting in a loss of cell–cell and cell–matrix interactions can promote cancer cell invasion and migration [64]. For example, CEACAM6 overexpression promotes the migration of NSCLC by enhancing integrin expression [65–67]. In addition, up-regulation of adhesion molecules may contribute to lung metastasis enhanced by local infection/inflammation [68]. Dysregulated focal adhesion and ECM receptor could result in tumor progression [69–71]. In the present study, the cell adhesion molecules, focal adhesion, and ECM receptor interaction were the main pathways in the high-risk groups. Thus, the lower OS probability in the high-risk group may correlate with activated adhesion molecules, focal adhesion, and ECM. DNA methylation is an important type of epigenetic modification [72]. Hypermethylation or hypomethylation could cause the down-regulation or overexpression of target genes, which further regulate NSCLC tumorigenesis and progression [73]. The overall methylation levels of the five genes were lower in the high-risk group in the present study, which may also correlate with the lower OS probability in the high-risk group. Thus, interfering with the above-mentioned pathways and targets may facilitate novel therapeutic methods and thus improve prognosis.

Immune cells are an important part of the tumor microenvironment and play a critical role in tumor development [74]. Tumor-associated macrophages and intra-tumoral CD8^+^ T cells are significantly associated with a poor prognosis in lung cancer [75–78]. Treg cells could promote lung cancer progression and metastasis [79–82] and are significantly associated with worse OS [83]. The neutrophil count in peripheral blood is an effective diagnostic biomarker for lung cancer [84]. Our research showed that high risk score was associated with increased macrophages M_2_, memory B cells, memory CD4^+^ T cells, neutrophils, and Treg cells. Thus, the high-risk group had a lower OS probability due to increased immune infiltration and may have a better treatment response to immunotherapy. Currently, immune checkpoint inhibitors (ICI) are becoming the standard first-line treatment for advanced NSCLC [85–87], and *PD1, PDL1*, and *CTLA4* are mainly targets for ICI [88–92]. The high-risk score was correlated with increased relative expression levels of immune checkpoint genes (*PD1, CLTA4*). Thus, the high-risk group may be more sensitive to immune checkpoint inhibitors against *PD1* and *CLTA4*. Most patients with lung cancer are diagnosed in an advanced stage [93]; thus, chemotherapy still serves as an important therapeutic method for them [94]. The high-risk group has a lower IC50 for six common chemotherapy drugs for lung cancer, which may be a reference option for chemotherapy drugs.

Highlights of the present study include the prognostic signature, which was constructed in the training cohort and validated in the test cohort, entire TCGA cohort and GSE30219, GSE157010, GSE73403, and GSE4573 datasets; and immunotherapy and chemotherapy response, which were identified to guide individualized treatment. In addition, the results of bioinformatic analysis were validated using real-time PCR in another cohort. Limitations of the present study are that our results were based on TCGA database and, thus, the prognostic signature needs to be validated in a clinical patient cohort. Moreover, the molecular mechanisms and specific role of ferroptosis-related genes, such as on lipid reactive oxygen species and ferrous ion accumulation, in LUSC need to be explored in further study.

## Conclusions

A ferroptosis-related gene signature (*ALOX5, TFRC, PHKG2, FADS2, NOX1*) was constructed in the present study. The OS probability was significantly lower in the high-risk group than in the low-risk group (*P*<0.001), and AUC value was 0.739. The high-risk score was associated with increased immune infiltration, lower methylation levels, higher immune checkpoint genes expression level, and better chemotherapy sensitivity. Therefore, a ferroptosis-related gene signature was successfully constructed to predict prognosis for LUSC, and it may facilitate novel therapeutic methods and guide individualized therapy including immunotherapy and chemotherapy.

## Data Availability

The data used to support the findings of the present study are available from TCGA database (https://portal.gdc.cancer.gov/) and GEO datasets (https://www.ncbi.nlm.nih.gov/gds/).

## Abbreviations

*ALOX5*: arachidonate 5-lipoxygenase
DEG: differentially expressed gene
ECM: extracellular matrix
*FADS2*: fatty acid desaturase 2
FC: fold change
GSEA: gene set enrichment analysis
HR: hazard ratio
ICI: immune checkpoint inhibitor
LUAD: lung adenocarcinoma
LUSC: lung squamous cell carcinoma
*NOX1*: NADPH oxidase 1
NSCLC: non-small cell lung carcinoma
OS: overall survival
PCA: principal component analysis
*PHKG2*: phosphorylase kinase catalytic subunit gamma 2
SIRT1: sirtuin 1
*TFRC*: transferrin receptor
TLR4: toll-like receptor 4
t-SNE: t-distributed stochastic neighbor embedding
